# Supplementary material for: Electrochemical Oxidation of Landfill Leachate after Biological Treatment by Electro-Fenton System with Corroding Electrode of Iron
Source: Int J Environ Res Public Health. 2022 Jun 24;19(13):7745. doi: 10.3390/ijerph19137745 (PMC9265374; doi:10.3390/ijerph19137745)
Supplement: Supplementary file 1 [file ijerph-19-07745-s001.zip › ijerph-1757144-supplementary.pdf]

Supporting Information

Electrochemical Oxidation of Landfill leachate after Biological Treatment by Electro-Fenton System with Corroding Electrode of Iron

Juan Tang[a], Shuo Yao[b], Fei Xiao[b], Jianxin Xia[a], Xuan Xing[1]*

[a] Department of Environmental Science, College of Life and Environmental Science, Minzu University of China, Beijing, 100081
[b] China Energy Conservation and Environmental Protection Group

Number of Pages (including this cover sheet): 5
Number of Table: 4

[1] *Corresponding author. Tel.: 010-6893 3621.

E-mail: xingxuanpku@163.com. (X. Xing)

Table S1 ANOVA test for response function Y_{COD}

Source	Sum of squares	Degree of freedom	Mean square	F-value	Prob＞F
Model	1925.66	9	213.96	130.62	＜0.0001
A	408.98	1	408.98	249.68	＜0.0001
B	1450.18	1	1450.18	885.32	＜0.0001
C	28.39	1	28.39	17.33	0.0042
AB	10.89	1	10.89	6.65	0.037
AC	0.0009	1	0.0009	0.00055	0.98
BC	1.01	1	1.01	0.62	0.46
A^2	0.006487	1	0.0065	0.0040	0.95
B^2	0.085	1	0.085	0.052	0.83
C^2	26.17	1	26.17	15.98	0.0052

Table S2 ANOVA test for response function $Y_{NH3\text{-}N}$

Source	Sum of squares	Degree of freedom	Mean square	F-value	Prob＞F
Model	4048.02	9	449.78	32.70	＜0.0001
A	783.29	1	783.29	56.96	0.0001
B	2882.30	1	2882.30	209.58	＜0.0001
C	123.48	1	123.48	8.98	0.02
AB	45.02	1	45.02	3.27	0.11
AC	0.029	1	0.029	0.0021	0.96
BC	13.36	1	13.36	0.97	0.36
A^2	25.09	1	25.09	1.82	0.22
B^2	106.48	1	106.48	7.74	0.027
C^2	77.00	1	77.00	5.60	0.050

Table S3 m/z table for initial landfill leachate analysis by GC-MS

Retention Time (min)	Detected ions m/z (% abundance)	Molecular Weight	Molecular Structure
10.07	45.2(100)·72.1(40)·89.0(12)	222	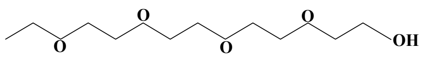
11.34	45(100)·59(47)·58(6)	162	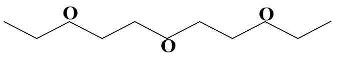
11.86	123(100)·95(49)·48(21)	282	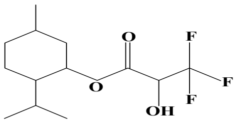
12.04	83(100) 84(30) 141(7)	155	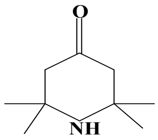
14.40	123(100) 109(48) 182(21)	268	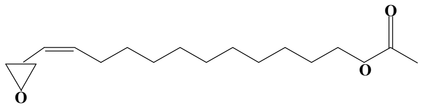
14.64	114(100) 57(63) 127(19)	129	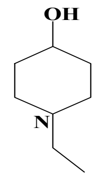
15.79	133(100) 77(56) 125(18)	245	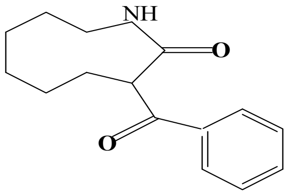
16.34	109(100) 57(18) 169(9)	226	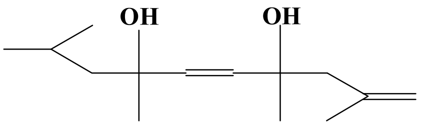
16.42	160(100) 132(33) 77(19)	175	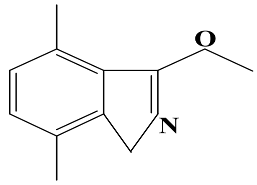
16.77	191(100) 57(35) 206(18)	206	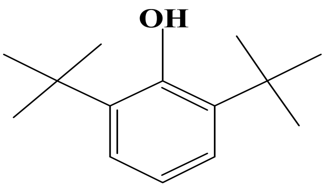

17.11	177(100) 220(57) 149(43)	220	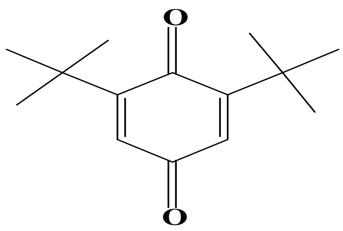
17.63	57(100) 55(23) 157(13)	211	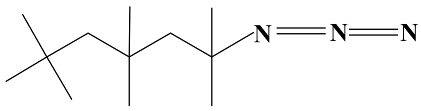
17.75	55(100) 110(32) 169(8)	182	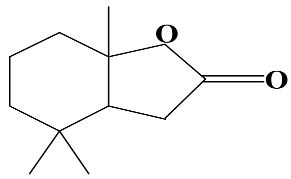
18.37	167(100) 110(25) 166(14)	224	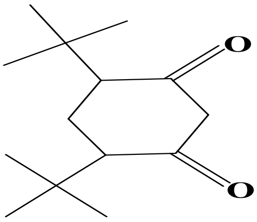
18.72	69(100) 135(48) 211(20)	270	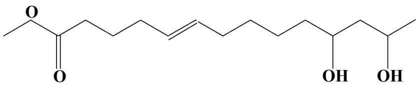
20.14	91(100) 154(48) 207(15)	310	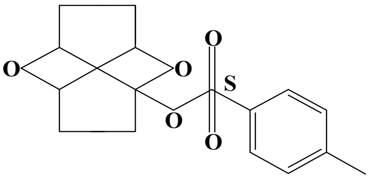
20.62	55(100) 180(60) 219(22)	235	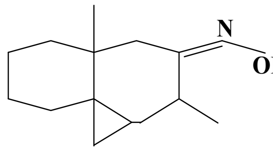
20.93	124(100) 55(35) 276(14)	430	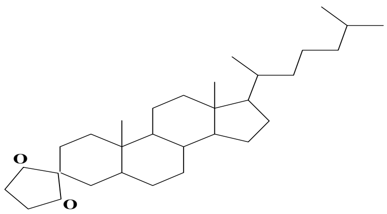
21.51	249(100) 55(40) 221(20)	324	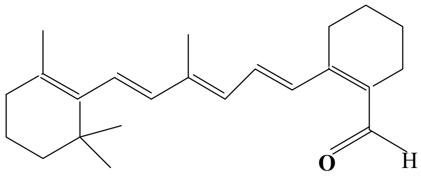

21.99	235(100) 57(37) 207(30)	250	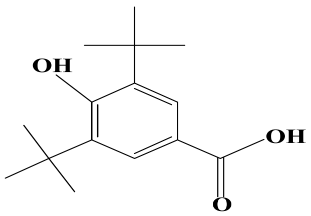
27.42	55(100) 83(48) 136(30)	294	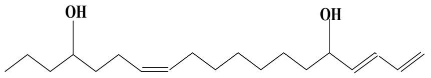
27.72	148(100) 57(72) 167(44)	536	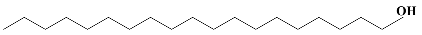
29.67	59(100) 69(20) 126(18)	337	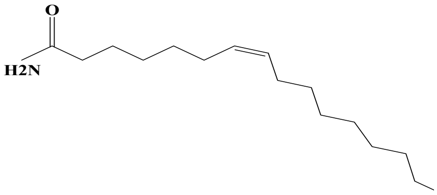

Table S4 m/z table for processed landfill leachate analysis by GC-MS

Retention Time (min)	Detected ions m/z (% abundance)	Molecular Weight	Molecular Structure
4.85	45(100)·48(49)·85(20)	84	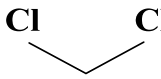
9.40	72(100)·116(41)·56(8)	116	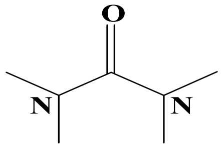
10.44	48(100)·112(37)·50(31)	148	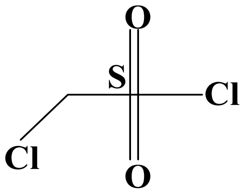
11.26	45(100) 72(45) 48(6)	162	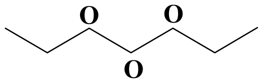
11.42	121(100) 77(21) 136(9)	180	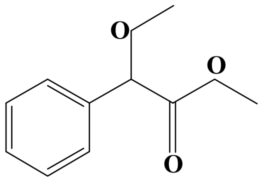
11.88	82(100) 47(19)	118	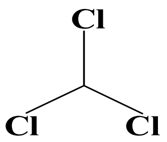
11.98	83(100) 84(31) 141(8)	155	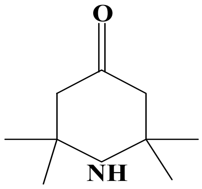
12.40	48(100) 50(35) 112(7)	148	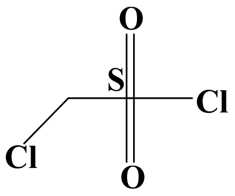
14.64	114(100) 57(55) 127(20)	171	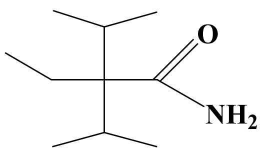

17.12	177(100) 220(55) 67(45)	220	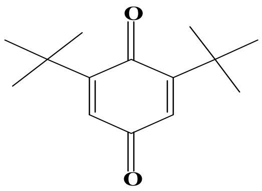
18.36	57(100) 110(25) 169(5)	224	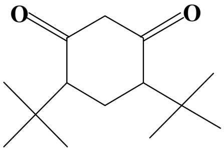
27.44	55(100) 83(53) 136(35)	266	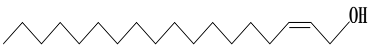
27.72	148(100) 57(44) 167(40)	487	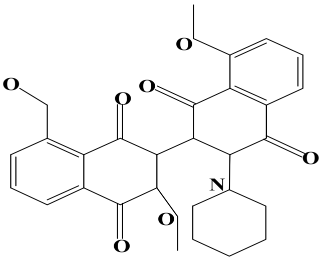
28.01	59(100) 57(35) 126(22)	281	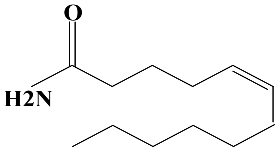
